# Energy-efficient quad tree-based clustering using edge-assisted UAV-relay to enhance network lifetime in WSN

**DOI:** 10.1038/s41598-024-68085-4

**Published:** 2024-07-26

**Authors:** K. H. Vijayendra Prasad, P. Sasikumar

**Affiliations:** School of Electronics Engineering (SENSE), VIT University, Vellore, Tamilnadu 632014 India

**Keywords:** WSN, Quad tree-based network, Clustering, UAV nodes, Engineering, Mathematics and computing

## Abstract

Wireless sensor networks' most prominent concern is energy optimization. It faces significant problems like high energy consumption, data loss, delay, and low network lifetime. To improve, it uses clustering. However, during clustering, coverage holes are most likely to appear near the network's edge, within the cluster, and between clusters. As a result, there are more energy holes and dead nodes; therefore, the goal of this work is to maximize node network lifetime and minimize energy consumption during data transmission in the wireless sensor network (WSN). The proposed work includes three entities: sensor nodes, an edge-assisted unmanned aerial vehicle (UAV), and a base station. It uses an edge-assisted unmanned aerial vehicle to provide additional resources to the UAV, which helps reduce energy consumption during data transmission. This research proposes using communication to enhance the speed and bandwidth of data transmission and reduce transmission latency. This work attempts to improve performance by increasing throughput.

## Introduction

Wireless Sensor Network (WSN) aims to monitor its area of interest and provide services for the Internet of Things (IoT)^1^. In WSN, cluster formation has proven to be a useful method for lowering sensor node energy usage, which improves the efficient utilization of bandwidth by reducing the transmission of redundant messages between the sensor node nodes. The devices can be vulnerable to privacy breaches^2^, as they can be used to track people's locations. The Cluster Head (CH) performs the data aggregation method, providing energy efficiency for sensor node nodes. In most cases, while clustering, data is sent directly from Cluster Members (CMs) to the CH^3^, which then aggregates the data and transmits it to the base station. The network's overall lifetime is shortened by data aggregation, which also increases the energy consumption of sensor nodes.

Sensor nodes reduce delay time in computation, which enhances the efficiency and effectiveness level of UAVs^4^. WSN includes efficient clustering and duty cycling to minimize energy consumption and maximize the network’s lifetime. It mainly performs duty cycling statically using various protocols and deep reinforcement learning by considering three states of sensor nodes: sleep, Active, and Transmit. In order to avoid high energy consumption, the sensor nodes are made inactive at certain time intervals^4^. However, various problems occurred due to performing duty cycling in a static manner that did not efficiently satisfy the objective. Multiple types of scheduling used in WSNs are cluster-based and priority-based.

The coverage hole is another problem affecting WSNs' energy efficiency. Lack of efficient coverage hole detection increases the probability of data retransmission, transmission rate decreases, and energy consumption. To improve the data transmission rate and avoid retransmission, The coherent approach^5^ tried to perform routing and detection of coverage holes and recovery based on clustering in WSN-IoT WSN carries out coverage hole detection and recovery. After cluster formation, the selection of the cluster head step was performed based on calculating the weights of the nodes by considering parameters such as distance and residual energy. Maintenance of cluster-by-cluster merging and cluster splitting was performed using the entropy function when the cluster size was large. Consider the locations within and among the clusters and the network edge location for detection of coverage hole and recovery of coverage hole by the hole manager using fuzzy logic. Finally, optimal selection of multi-hop route using multi-objective emperor penguin optimization algorithm (MO-EPO) for efficient routing, However, various challenges are present in coverage hole detection and recovery. A poor selection of sensor node nodes to repair the coverage hole results in inefficient repair, reducing network lifetime. Various deep learning approaches to improve the network lifetime.

However, the value of k must be pre-defined in this algorithm, which decreases the stability of the cluster and affects the performance of the network cluster head selection by considering residual energy and distance for weight calculation. However, these parameters are not enough to select the cluster head efficiently, Cluster members performed coverage hole detection by considering the sensing radius. However, the lack of efficient transmitting power in cluster members increases the latency for detection of coverage holes; hole managers are used to recover the cover holes by Fuzzy logic. However, it needs high processing time and provides inaccurate results. In Table 1, the nomenclature of the work is included.

**Table 1:** Nomenclature table.

Acronym	Term
WSN	Wireless sensor network
UAV	Unmanned aerial vehicle
CH	Cluster head
CM	Cluster member
BS	Base station
CHSRA	Cluster head selection and rotation algorithm
QoS	Quality of service
OABC	Oppositional artificial bee colony
OPTIC	Optimal clustering
BER	Bit error rate
IUKF	Improved unscented Kalman filter
TA-TD3	Twin agent twin delay deep deterministic algorithm
CMO	Cat and mouse optimization algorithm
DDPG	Deep deterministic policy gradient
TD3	Twin delayed deep deterministic
HWSN	Heterogeneous wireless sensor network
MOEPO	Multi-objective emperor penguin algorithm
IoT	Internet of things
PDR	Packet delivery ratio

### Motivations & objectives

To improve the sensor node's performance, the following challenges are observed, impacting energy consumption.

- Instability in clustering: In many studies, cluster formation and cluster head selection were used to boost data transmission and lower energy consumption. However, poor cluster management and inefficient cluster head selection increase the complexity, leading to low cluster stability and reliability.
- Low network lifetime: Some previous works performed sleep scheduling and coverage hole recovery to minimize the sensor nodes' energy consumption. However, ineffective scheduling by considering insufficient parameters and poor selection of sensor nodes for coverage hole recovery does not meet the objective of the process that reduces the network's lifetime.
- High data loss: Relay selection was performed for efficient data transmission with a high delivery ratio, but lack of sufficient transmitting power in the relay node reduced the efficiency of data transmission, leading to high data loss and selection of cluster head with insufficient parameters also increases the data loss
- Poor relay selection: Relay nodes were selected to increase the data transmission rate with low latency and energy consumption. However, insufficient parameters (i.e., location and velocity) were used to perform relay selection, and the small coverage area of the relay node required multiple hops to reach the base station, increasing the transmission delay and consuming more energy for data transmission.

The objective of this research is to reduce data loss and delay in WSN while maximizing energy efficiency and network lifetime. It also addresses high energy consumption, high data loss, high delay, and low network lifetime in WSN. This research aims to perform.

- Quad tree-based clustering to form clusters by splitting the zone into four quadrants based on the density of the nodes present in the network to improve the network's performance.
- Multiple parameters, such as residual energy and link stability, are considered for optimal CH selection to collect the data from the CMs and transmit it to the base station with a high packet delivery rate. Here,
- It performs coverage hole detection and recovery using a twin agent-based twin delay deep deterministic algorithm (TA-TD3) with a high sensing radius. Repairing coverage holes is accomplished by selecting optimal nodes and considering parameters such as lifetime and coverage level, which increases the accuracy of coverage hole detection and recovery and improves the network lifetime.
- Finally, the UAV nodes transmit the data to the base station. The CMO(Cat and mouse) optimization algorithm is used to select the optimal UAV node. Unmanned aerial vehicles (UAVs) are increasing in demand due to their importance in providing robust and reliable communication systems for many civilian and military domains^6^.

The following portions of this study are organized as follows: "Related works" explains prior research and any gaps in that research. "System model" describes the research technique in detail and is illustrated with examples of the procedure, algorithms, and mathematical representations. "Simulation analysis" includes the simulation setup and comparison analysis, and "Impact of delay" describes this work's research summary and experimental findings. The conclusion of the suggested study and future directions are presented in "impact of reliability".

## Related works

This paper analyzes many proposals for reducing energy consumption in WSNs. Clustering has always played a crucial role in the energy optimization of wireless sensor node networks**, so** some of the recent works are analyzed here.

Article^7^ proposes an energy-efficient protocol for improving the network lifetime of the WSN-IoT networks. The proposed work includes three phases: cluster selection, cluster balance, and CH selection. Initially, it performs an optimal number of cluster selections, which the base station implements. The formation of balanced clusters is then suggested using modified fuzzy C-means clustering. The CHSRA back-off timer technique is used to pick the CH after cluster formation is complete. Here, a dynamic threshold is used to balance the energy consumption of CH through the rotating process. In this case, the selection of the CH is based on residual energy and distance, which are insufficient to choose an ideal CH without compromising the network's stability and dependability.

The article^8^ suggests using fuzzy-based clustering to increase WSN network longevity. This study first introduces the energy model, and then it uses a fuzzy technique to cluster nodes based on their distance, energy, and degree. Following clustering, node energy, node concentration, and centrality are taken into account when choosing the CH. This study expanded network lifetime and decreased energy usage in this way. The experimental outcome demonstrates that the suggested work achieved better network lifespan performance and energy efficiency. Here, fuzzy rules are used to generate clusters while taking into account a small number of metrics, which is insufficient for stable clustering. Furthermore, fuzzy logic never yields an ideal solution, which causes clustering instability.

In the article^9^, a hybrid metaheuristic method for cluster-based routing in WSN is proposed. Here, clustering is done by a hybrid optimization algorithm, including brainstorming optimization and levy distribution algorithm. After completing cluster construction, Based on network demand, energy, and distance, CHs are chosen. The Water Wave Optimization and Hill Climbing methods are used for selecting the optimal set of routes between CH and the base station by considering energy and distance. The data are transmitted from CH to the base station based on the optimal path. The experimental result shows that the suggested work provides superior performance in terms of packet delivery ratio, packet loss rate, end-to-end delay, and network longevity and efficiency. Here, optimal routes are selected between CH and the base station by choosing a static node as a relay, which leads to instability in routing, and it also consumes much energy to select the optimal path by choosing a number of next hops.

In the article^10^ it proposes dynamic scheduling to improve energy efficiency in WSN. For that purpose, this research proposed content-based dynamic scheduling using a two-way communication model. Active-live state, sleep-live state, active-sleep state, and sleep-sleep state are its four energy states. This approach comprises two types of communication, forward communication, and backward communication, based on these energy states. Therefore, the results of the simulation demonstrate that the suggested work performed better in terms of energy efficiency, stability, and network longevity.

In the article^11^ it proposes adaptive duty cycling for reducing energy consumption in WSN. Here, QoS-aware duty cycling is proposed by considering priority and queue length. Duty cycling helps sensor nodes wake up and sleep at the appropriate times to save energy and lengthen the network's lifetime. In this case, the average value of the queue length and priority is used to modify duty cycling. In the end, a network simulator (NS-3) simulates several traffic scenarios. The trial outcome demonstrates that, in comparison to previous methods, the suggested work performed better in terms of lifetime and energy efficiency. Duty rotation is modified here in accordance with queue length and priority. This, however, needs to be improved for the best scheduling. In addition, inadequate scheduling brought on by a lack of historical data shortens the lifetime of the network.

In edge-enabled WSN-IoT networks, the paper^3^ suggests cluster-based routing via an optimization technique. Two procedures are involved in the proposed work: routing and clustering. Black widow optimization is used for clustering, and residual energy, link quality, communication cost, neighbor count, node marginality, and restart value are taken into account for CH selection. The Oppositional Artificial Bee Colony (OABC) algorithm then handles routing in order to address the energy reduction issue. The outcomes of the simulation show that the suggested work performed better in terms of packet delivery ratio, network lifetime, energy efficiency, and packet loss rate. The OABC algorithm, which chooses non-optimal routes with high time and energy consumption, has a high computational complexity and a slow convergence rate when it comes to routing.

The article^12^ proposes a novel clustering algorithm to increase network lifetime in WSN. The network model, energy model, path loss model, and network longevity model are among the four stages of the proposed study. The proposed OPTIC clustering method selects the optimal CH for reducing message overhead, which divides the CH states into four such as on, action CH, CH, and MN. It increased the stability of clustering and increased network lifetime. In comparison to conventional clustering methods, the simulation result demonstrates that the suggested clustering algorithm obtained great performance in terms of network lifetime, end-to-end delay, and throughput.

The article^13^ proposes coverage hole repairing for balancing the energy consumption of the nodes in IoT. Here, it is suggested that mobile edge computing is used to patch coverage holes, therefore increasing network lifetime and energy efficiency. It is solved by proposing energy energy-balanced dispatch algorithm that controls the mobile edge nodes moving at shorter distances by updating the bounding values of the coverage hole. Here, a bipartite graph is constructed by evaluating the relationship between the mobile edge nodes. Finally, The experimental outcome demonstrates that the suggested work performed better in terms of energy efficiency and network longevity. Here, mobile edge nodes are used to perform coverage hole repairing. However, they cover less area, which degrades the performance of coverage hole detection and repair.

The article^14^ uses fuzzy logic in WSN to do clustering by splitting the network into many equal-sized zones. The zone monitor is chosen based on the highest energy node, and the remaining energy and distance are taken into account when selecting the CH. The equal CH distribution, uniform clustering, and unequal clustering were all accounted for by this study. By performing clustering based on a minimum CH threshold, the network lifetime is extended, and the energy consumption is decreased. CH selection and clustering are done using fuzzy logic. In this case, normalization is carried out to raise performance by normalizing the fuzzy inputs. The simulation's outcome demonstrates that, in comparison to other cutting-edge techniques, the suggested work performed better. Here, zone-based grouping is carried out using the fuzzy technique. But it could yield a better outcome.

This article^15^ proposes a dynamic node scheduling algorithm to control energy utilization and enhance network lifetime in WSNs. For scheduling, this research proposed an optimized backoff sleep protocol method that schedules the nodes into three states such as active, sleep, and probe. In which the sleep node turns off its transceiver to reduce energy, and the probing node transmits the hello packets within its coverage. Active node continuously senses and transmit the sensed data to the other nodes in the environment. The results of the simulation indicate that the proposed work produced higher performance energy. Delay, packet loss rate, and packet reception rate. Here, dynamic node scheduling is performed by optimized backoff sleep protocol; however, it provides poor scheduling due to a lack of historical information on the sensor nodes. The following Table 2 summarize the existing works.

**Table 2:** Summary of the existing works.

Article	Approach	Key techniques	Key findings	Limitations
^7^	Energy-efficient protocol	Cluster selection, cluster balance, CH selection	Improved network lifetime and energy efficiency	Insufficient metrics for stable clustering, compromise on network stability
^8^	Fuzzy-based clustering	Energy model, fuzzy clustering based on distance, energy, and degree	Increased network longevity, decreased energy usage	Insufficient metrics for stable clustering, fuzzy logic limitations
^9^	Hybrid metaheuristic method	Hybrid optimization algorithm for clustering, Water Wave Optimization, and Hill Climbing for route selection	Superior performance in packet delivery, loss rate, end-to-end delay, network longevity	Instability in routing due to static relay selection, energy consumption in selecting optimal paths
^10^	Dynamic scheduling	Content-based dynamic scheduling with a 2-way communication model	Improved energy efficiency, stability, and network longevity	NA
^11^	Adaptive duty cycling	QoS-aware duty cycling considering priority and queue length	Improved lifetime and energy efficiency	Inadequate scheduling due to lack of historical data
^3^	Cluster-based routing via optimization	Black widow optimization for clustering, Oppositional Artificial Bee Colony (OABC) for routing	Better performance in packet delivery, network lifetime, energy efficiency	High computational complexity and slow convergence rate in routing
^12^	Novel clustering algorithm	OPTIC clustering method for CH selection, network, and energy models	Increased stability, improved network lifetime	NA
^13^	Coverage hole repairing	Mobile edge computing, energy-balanced dispatch algorithm	Improved energy efficiency and network longevity	Degrades performance due to less coverage area by mobile edge nodes
^14^	Fuzzy logic-based clustering	Zone-based clustering using fuzzy logic, CH selection based on energy and distance	Extended network lifetime, decreased energy consumption	The zone-based grouping may not yield the optimal outcome
^15^	Dynamic node scheduling	Optimized backoff sleep protocol for node scheduling	Higher performance in energy efficiency, delay, and packet loss rate	Poor scheduling due to a lack of historical information

## System model

The objectives of this work are to maximize sensor network longevity and minimize energy use. (nodes) (i.e., static and mobile) during data transmission in WSN.Three entities are included in the proposed work: sensors, edge-assisted UAVs, and base stations. The edge-assisted UAV is used to provide additional resources to the UAV, which helps to reduce energy consumption during data transmission. This research tries to communicate in order to enhance the speed and bandwidth in data transmission and reduce transmission latency. explains the flow of the proposed work. And also the Table 3 summarizes of different variables used in this article.

**Table 3:** Summary of system variables.

Symbol	Description
${\text{E}}_{\text{tx}}$	Transmit energy
${\text{E}}_{\text{rx}}$	Receiver energy
d	Distance
k	Proportionality constant
(${E}_{elec}$)	Electronics energy
$({E}_{fs})$	Free space energy
$({E}_{amp})$	Energy of multi-path
$N$	Number of nodes
$D$	Density
$A$	Area of the deployment region
$({D}_{min})$	Minimum desired node density
$({N}_{l})$	Number of nodes in each level
$({E}_{i})$	Residual energy
$({C}_{i})$	Expected coverage rate
${B}_{i}$	Buffer factor
*H*	Historical data matrix
${x}_{k|k-1}$	Predicted state vector
${P}_{k|k-1}$	Predicted error covariance matrix
${F}_{k-1|k-1}^{T}$	state transition matrix
$\text{you }{u}_{k}$	Control input
${z}_{k}$	Measurement matrix
*h(.)*	Measurement function
${v}_{k}$	Measurement noise
*R*	Measurement of the noise covariance matrix
${H}_{k}$	Kalman noise
${y}_{k}$	Actual measurement
*Q*	Process noise covariance matrix
R(t)	Reward function
$\alpha \& \beta$	Weighting factors
${Q}_{TD3}$	Q value estimated by TD3 algorithm
${P}_{m}$	Final position
*f(u)*	Objective function
$\zeta \text{and} \eta$	Weighting factors
${E}_{c}$	Energy consumption
${N}_{i}$	Initial energy
${E}_{r}$	Remaining energy
${P}_{R}$	Received packets
${P}_{t}$	Transmitted packets
$Tr$	Throughput
${S}_{t}$	Amount of successfully transmitted packets
${D}_{L}$	Delay
${C}_{c}$	Current completion time
${C}_{ex}$	Expected completion time
${P}_{c}$	Percentage of coverage
${C}_{a}$	Coverage area
${T}_{a}$	Total area

The proposed work includes four consecutive phases, which are defined as follows,

1. Quadtree based clustering
2. Dynamic duty cycling
3. Energy efficient coverage hole detection and recovery
4. Optimal UAV-relay selection.

The following Table 4 indicates the goals of the proposed work.

**Table 4:** Design goals of the proposed model.

Processes	Algorithms	Goals
Quad tree-based network construction	–	(i) Improved network management
Dynamic duty cycling	IUKF	(i) Energy consumption is reduced (ii) Network lifetime enhancement
Coverage hole detection and recovery	TA-TD3	(i) Two agents are used, one for coverage hole detection and one for recovery
Optimal UAV relay selection	CMO	(i) Reduces delay in transmission (ii) Improves overall energy efficiency

### Radio model for communication

In a wireless sensor network, the first-order radio model is a simplified abstraction which is having a behavior to communicate. It represents the energy consumption and transmission range of these devices. The first-order radio model has two key parameters, like

1. Transmit Energy (${\text{E}}_{\text{tx}}$): This represents the energy consumed by a node to transmit data to a certain distance. It expands the energy based on the distance to the receiver
2. Receiver Energy (${\text{E}}_{\text{rx}}$): This represents the energy consumed by the node to receive certain data from another node. Like the transmitter, it depends on the distance between the sender and receiver.

The first-order radio model offers an evaluation of energy consumed when transmission or reception is made by a sensor node at each cycle. The energy consumed during the transmission and reception is typically proportional to the distance covered. Hence, the energy required to transmit or receive a packet over distance ‘d’ can be represented as

1 $${\text{E}}_{\text{tx}}= \text{k*d} $$

2 $${\text{E}}_{\text{rx}}= \text{k*d} $$

**Figure 1:**
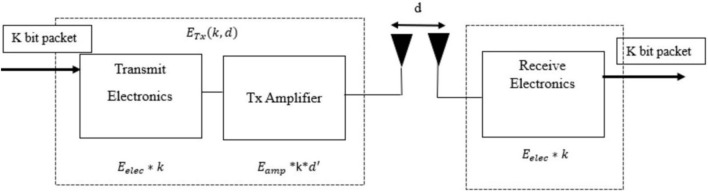
Radio model for communication.

In Fig. 1, The basic concept of radio energy is illustrated. In this case, the link between energy consumption and distance is represented by the proportionality constant "k." In the early phases of network design and analysis, this model can be applied to basic simulations and optimizations in networks. In wireless sensor networks, first, examine and comprehend the sensor nodes' communication range and energy efficiency. Any transmitter's energy used to broadcast a k-bit message across a d-distance is,

3 $${\text{E}}_{\text{Tx}}\left(\text{k,d}\right)=\{\text{k*}{\text{E}}_{{\text{ele}}{\text{c}}}+\text{k*}{\text{E}}_{\text{fs}}\text{*}{\text{d}}\}^{2}\text{ d}{<}{\text{d0}}$$

4 $$\text{k*}{\text{E}}_{\text{elec}}+ \text{k*} {\text{E}}_{\text{amp}}\text{*}{\text{d}}^{4}\text{ d} \ge \, {\text{d0}}$$

5 $$\text{d}0=\sqrt{\frac{{\text{E}}_{\text{fs}}}{{\text{E}}_{\text{amp}}}} \, $$

The first term represents the energy required for radio dissipation, and the energy needed for radio amplification is defined by the second. The electronics energy (${{\varvec{E}}}_{{\varvec{e}}{\varvec{l}}{\varvec{e}}{\varvec{c}}}$) depends on factors such as the digital coding, modulation, filtering, and spreading of the signal, the use of free space (${{\varvec{E}}}_{{\varvec{f}}{\varvec{s}}}$) and the multi-path (${{\varvec{E}}}_{{\varvec{a}}{\varvec{m}}{\varvec{p}}}$) Fading channel models depend upon the transmission distance d.

### Communication energy model For UAV nodes

The communication energy model of UAVs typically represents the energy consumption associated with wireless data transmission and reception. This energy model includes various factors like data rate, transmission distance, transmission power, and communication protocol used. The energy consumed during data transmission can be represented using the following equation.

6 $${E}_{transmisson }={P}_{tx}*d*\frac{1}{Data Rate}$$

where ${E}_{transmisson}$ is the energy consumed during data transmission (in joules or Watt-seconds), and ${P}_{tx}$ is the transmission power (in watts), and d is the distance over which the data is transmitted(in meters). Data Rate is the rate at which data is transmitted (in bits per second).

**Figure 2:**
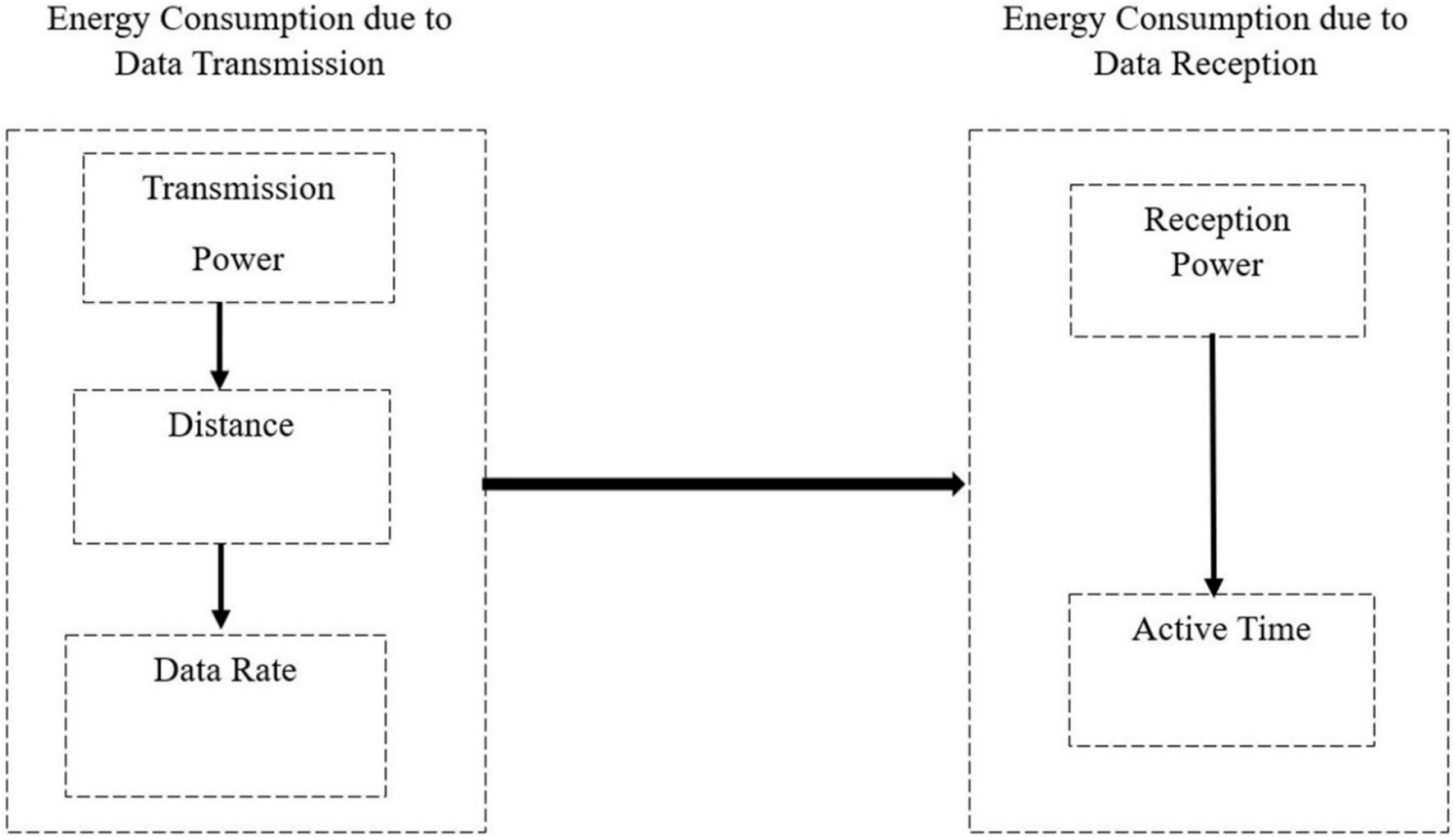
Communication energy model of UAVs.

The energy consumed during data reception can be represented as

7 $${E}_{reception }={P}_{rx}*{t}_{active}$$

where ${E}_{reception}$ is the energy consumed during data reception (in joules or watt-seconds), Where ${P}_{rx}$ is the power consumption(in watts)of the receiver.and ${t}_{active}$ is the active reception(in seconds). Some other factors are also considered when calculating communication energy consumption, such as idle energy consumption, Protocol Efficiency, Packet overheads, Dynamic Power levels, Data Aggregation and Duty Cycling, and the above Fig. 2 shows a simplified representation of the energy model.

### Quad tree-based clustering

**Figure 3:**
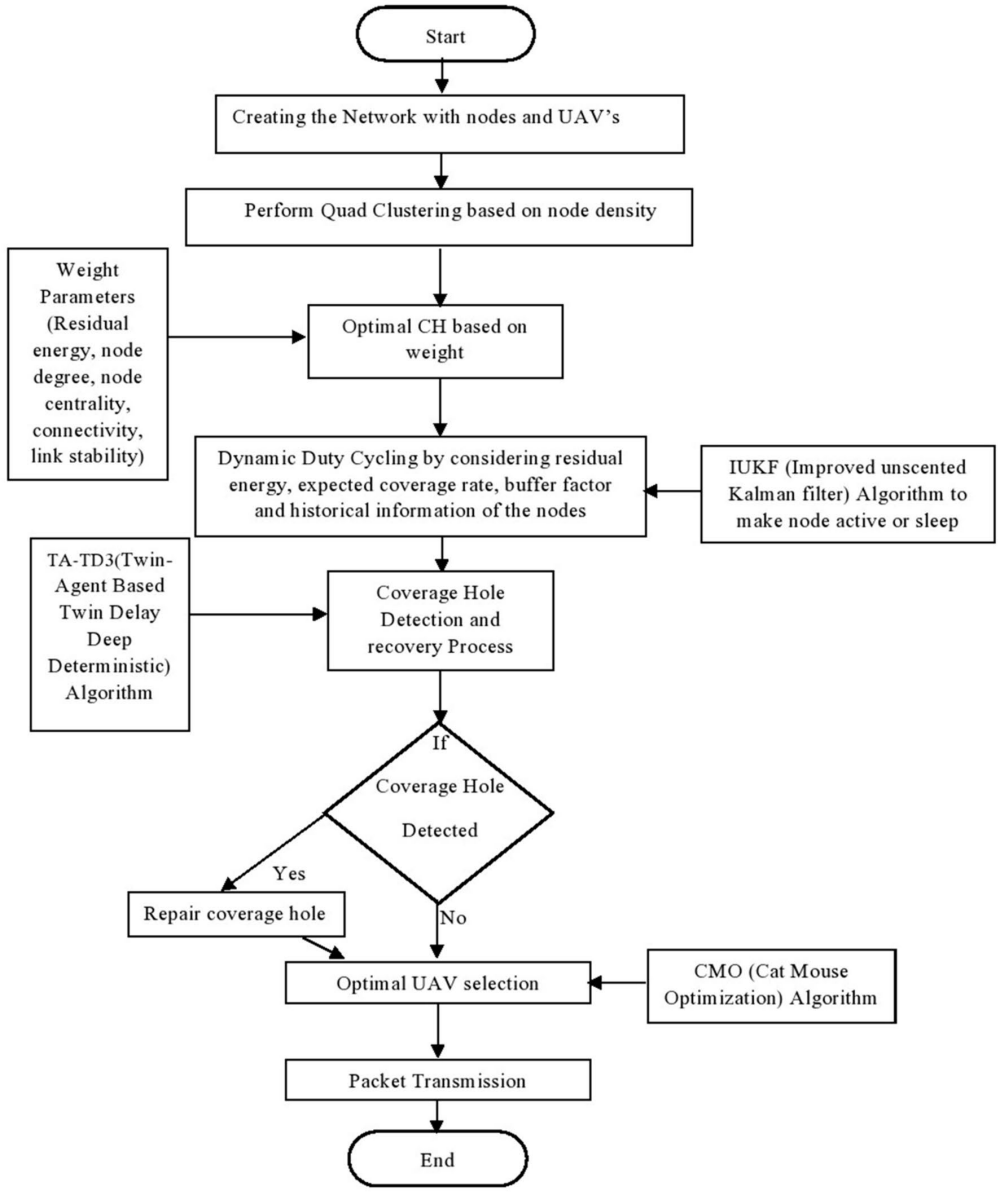
Flow chart of the operation of energy efficient quad clustering.

Here in this, a Quadtree algorithm for clustering is proposed in which the network is split into four Quad zones for clustering. Each zone is again divided into four Quadrants (four sections) based on the node density in the network, Fig. 3 represents the flow of the proposed model and this data structure was named a Quad Tree by Finkel and Bentley in 1974^16^, which improves the overall network performance. Quadtree clustering is a very popular and common technique that is used to manage the nodes efficiently. So here in this work, we are considering the simulation for 100 nodes. The network scenario can be explained as:

Let N be the total number of nodes used in this case, which is 100 (N = 100), A be the area of the region's deployment, and D be the node density, which is basically the number of nodes per unit area. Let Q be the quadtree structure.

So, first, it calculates the node density, which plays a key role in quad clustering. It is denoted as

8 $$D=\frac{N}{A}$$

Once the node density is derived, the Quadtree structure is designed where it splits the nodes into four equal quadrants, and each quadrant is further divided into sub-quadrants according to the requirement. In this case, the depth of the Quadtree can be determined by the node density, and the Quadtree subdivision can be explained as follows.

Let L be the depth of the Quadtree, and the number of sub-divisions can be determined using this formula.

9 $$\text{L}= [ {\text{log}}_{2}\left(\frac{A}{{D}_{min}}\right)]$$

Where ${D}_{min}$ is the minimum desired node density. This formula ensures that the node count does not exceed the ${D}_{min}$ After the nodes are split into quadrants, each level L can determine the level of the structure using the equation below.

10 $${N}_{L}=\frac{D}{{4}^{l}}.A$$

**Figure 4:**
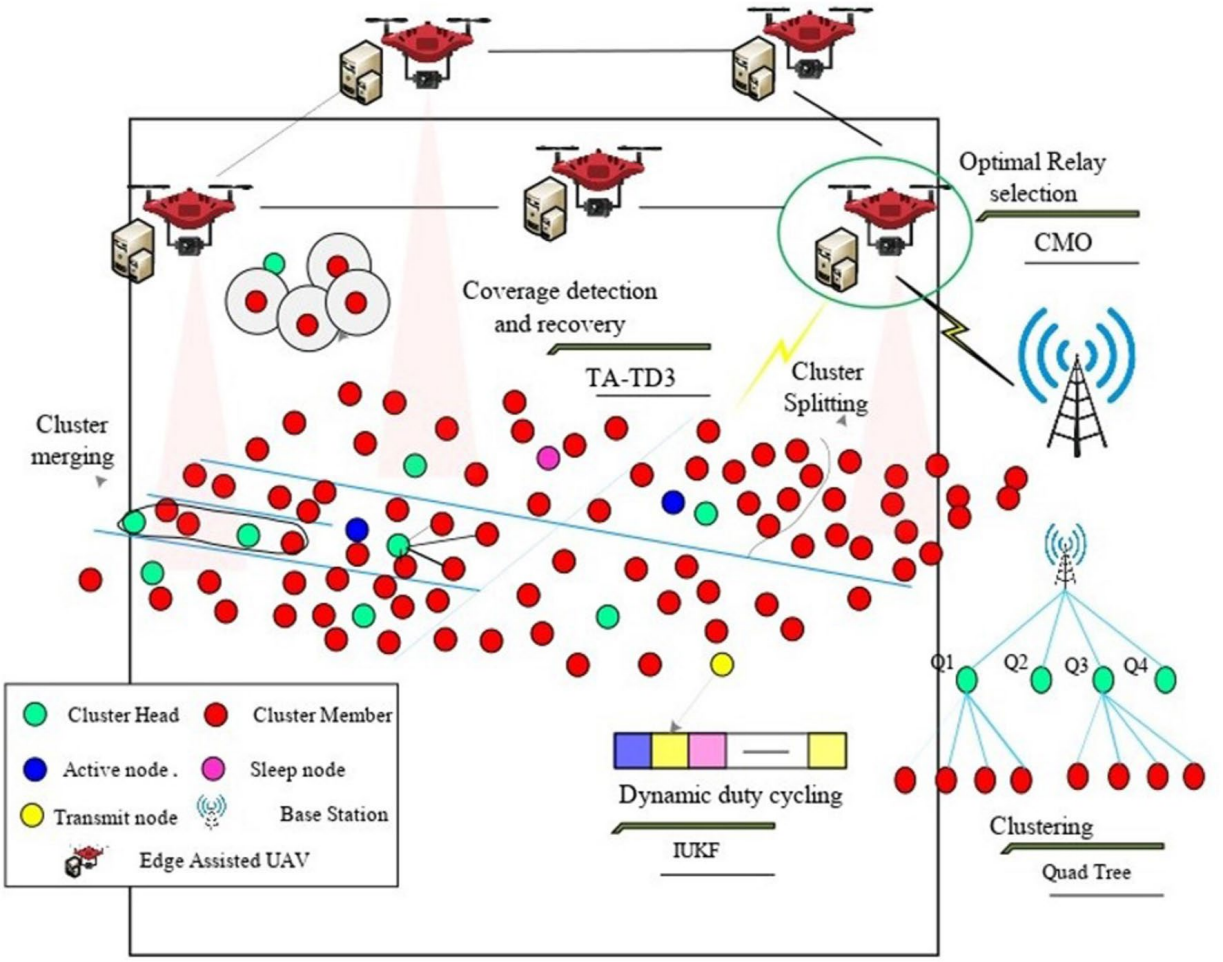
: Model of energy efficient quadtree based clustering model of energy efficient quad base clustering.

All the nodes can be calculated with the sum of the nodes at each level, which can be represented as

11 $${N}_{total}=\sum_{l=0}^{L}{N}_{l}$$

where the ${N}_{l}$ Is the number of nodes in each level? The major advantage of quadtree clustering is that it improves the storage and efficiency of the network, and it avoids unnecessary sub-divisions in regions with lower node density. Once clusters are formed, it performs optimal CH selection by calculating the weight values of residual energy, node degree, node centrality, connectivity, and link stability. The high-weight value node is selected as the cluster head (CH). Others are known as cluster members (CM). The CH is used to collect the data from the CMs and transmit the collected data to the base station (BS). The Cluster maintenance is an important process to maintain stability and reliability in communication. For that purpose, it performs cluster splitting and merging based on a threshold, which is calculated based on Cross-entropy by considering node density. In this cross-entropy clustering, the main goal is to minimize the cost function. The cost function can be calculated in two steps. First, it determines the parameters of the best Gaussian function in each cluster. Next, it builds a new division of X by adding points to the closest Gaussian density or cluster. This process is repeated until the cost function change is less than the predetermined threshold. If the node density is greater than the threshold value, then it performs cluster splitting, in which the CH selection is based on calculating weight values. If the node density is less than the threshold, then it conducts cluster merging, in which the CH selection is based on a comparison of these two CHs, which one has a high weight that is selected as the current CH. The following Fig. 4 explains the system model of the work.

**Figure:**
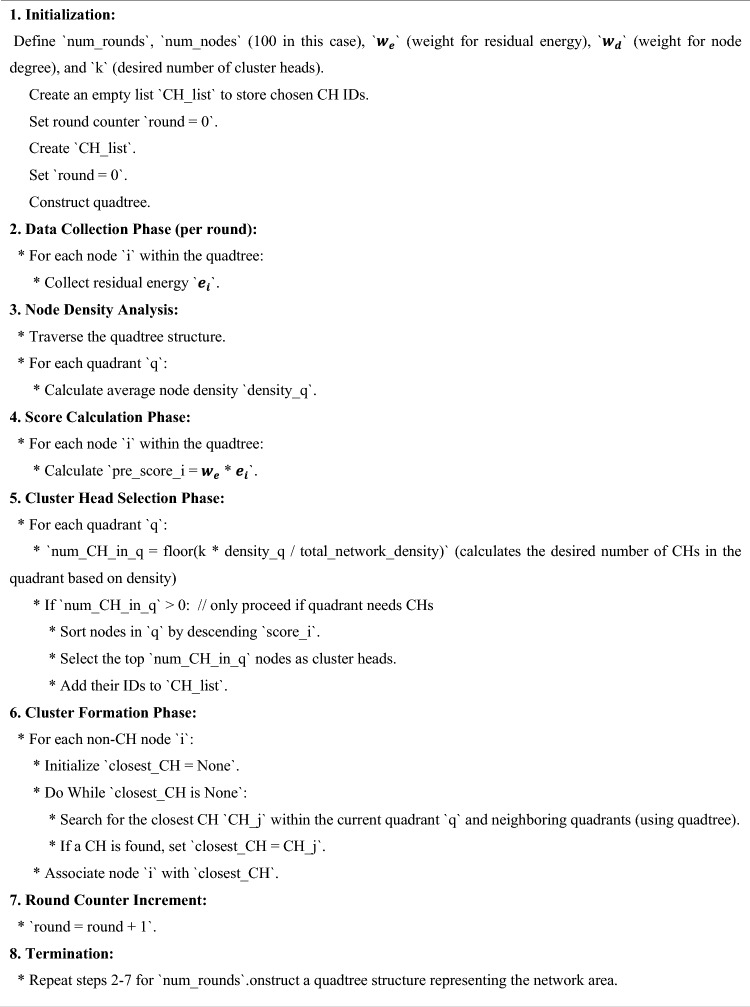
Algorithm for selecting optimal cluster heads in quad tree structure.

### Dynamic duty cycling

The proposed work uses dynamic duty based on the Improved Unscented Kalman Filter (IUKF) method to schedule the sensor nodes into transmit, active, and sleep states after quadtree clustering is completed to minimize energy usage. Remaining energy, anticipated coverage rate, buffer factor, and node history are taken into account while scheduling. In order to reduce the sleep latency and to balance energy consumption among sensor nodes. The system uses the duty cycle, whereby sensor nodes have independent duty cycles, to save energy. The residual energy to be considered for performing the duty cycling There are many factors involved during operation. Residual energy, expected coverage rate, buffer factor, and historical information of the nodes are considered for performing the Duty cycling. The residual energy in the node can be represented as ${E}_{i}$ Which is the residual energy of node *I. I*n general, the residual energy is the remaining energy that is available after performing a certain operation. The energy consumption can be represented as s

12 $${E}_{i}\left(t+1\right)={E}_{i}\left(t\right)-\text{Energy Consumed}(i,t)$$

Once the residual energy is calculated, then the expected coverage rate ${C}_{i}$ is to be calculated, which is used to find how the node is covering the designated area. This can be defined based on the historical data and the predicted data of the sensor measurements. Let's consider that the coverage data is affected by the residual energy and historical coverage data.

13 $${C}_{i}\left(t+1\right)=f({E}_{i}\left(t\right), Historical\, coverage\left(i\right))$$

Then, the buffer factor is calculated, which is represented as ${B}_{i}$ It is the time at which the node should be active compared to its actual potential time, and the expected coverage rate and the historical buffer factor can impact this. The following equation represents the buffer factor.

14 $${B}_{i}\left(t+1\right)=g({C}_{i}(t),\text{ Historical Buffer factor}(i))$$

The historical information of the data plays an important role in the coverage of buffer factors and other relevant parameters, which is useful in decision-making. Consider H as the historical data matrix. Each row represents a different time, and each column corresponds to a different node parameter, which can be expressed as follows.

15 $$H(t+1)=\text{UpdateHistoricalData}(H(t), {C}_{i}(t), {B}_{i}( t),$$

After calculating the above, the IUKF (improved unscented Kalman filter) is performed, which is a recursive estimation algorithm that combines predicted state estimates with measurements to provide state estimates over time. In this scenario, the state vector includes elements like residual energy, expected coverage rate, and buffer factor. The prediction and update steps are expressed as follows.

The first process of this step is the prediction step.

16 $${x}_{k|k-1}=f({x}_{k-1|k-1},{u}_{k})$$

17 $${P}_{k|k-1}=Q+{F}_{k-1|k-1}{P}_{k-1|k-1}{F}_{k-1|k-1}^{T}$$

Then it is followed by the update step

18 $${z}_{k}=h\left({x}_{k|k-1}\right)+{v}_{k}$$

19 $${S}_{k}=R+{H}_{k}{P}_{k|k-1}{H}_{k}^{T}$$

20 $${K}_{k}={P}_{k|k-1}{H}_{k}^{T}{S}_{k}^{-1}$$

21 $${P}_{k|k}={P}_{k|k-1}-{K}_{k}{S}_{K}{K}_{k}^{T}$$

where ${x}_{k|k-1}$ is the predicted state vector and ${P}_{k|k-1}$ is the predicted error covariance matrix ${u}_{k}$ is the control input, Q is the process noise covariance matrix, ${F}_{k-1|k-1}^{T}$ is the state transition matrix ${z}_{k}$ is the measurement *h(.)* is the measurement function ${v}_{k}$ is the measurement noise $R$ is the measurement of the noise covariance matrix and ${H}_{k}$ is the Kalman gain and ${y}_{k}$ is the actual measurement by the above steps, the IUKF algorithm is performed, and then the decision rule is taken based on the above-mentioned estimated factor ${x}_{k|k}$ It decides on duty cycling for each node. If, for instance, the expected coverage rate is low and the residual energy is also low, then the node is kept in sleep mode, whereas if the buffer factor is high, then the node might be kept in active mode for a longer duration. Duty cycling is performed using the process mentioned above.

### Energy-efficient coverage hole detection and recovery

Once the above two phases of operation are completed. This phase is used to detect and recover the coverage hole in the network. In this research, A Twin Agent twin-based twin Delay Deep Deterministic (TA-TD3) technique is used; this is built based on the Deep Deterministic Policy Gradient algorithm (DDPG), which helps in increasing stability and performance. It combines Policy Gradient, Actor Network, and Double Deep Q-learning—three potent Deep Reinforcement learning approaches. Coverage hole detection involves identifying regions where the expected coverage rate is much low than the desired value. The Twin Delayed Deep Deterministic (TD3) is a reinforcement learning algorithm that is used to learn policy and sequential decision-making. In this scenario, the TD3 algorithm is used to detect and repair coverage holes efficiently. Let's assume the TD3 policy as ${\pi }_{TD3}$ reinforcement involves an agent interacting with an environment to learn a policy that maximizes the reward function. In this scenario, the agent is the algorithm executing the TD3 policy, and the environment consists of the coverage holes and the sensor nodes. The reward function is an important component in reinforcement learning. It helps the agent’s behavior obtain the desired outcomes.

**Figure:**
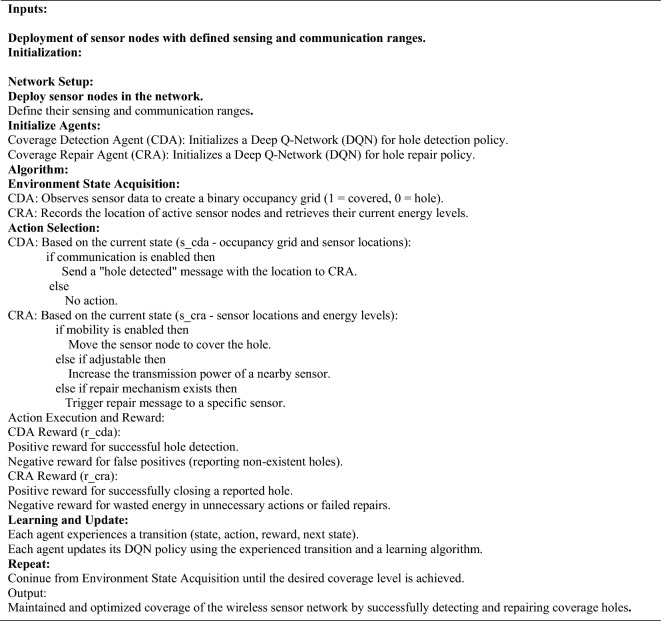
Algorithm for coverage hole detection and repair with two agents.

The Reward function in this scenario can be explained by the R(t), which considers factors like coverage improvement and energy consumption.

22 $$\text{R}(\text{t}) = \alpha .\text{ CoverageImprovement}(\text{t}) -\beta .\text{ Energy Consumption}(\text{t})$$

where $\alpha$ and $\beta$ are weighting factors, and CoverageImprovement(t) and EnergyConsumption(t) represent how much coverage has improved and how much energy is consumed, respectively, at every time step t. The TD3 algorithms aim to maximize the expected cumulative reward over time. The following step updates the policy.

23 $$ {\pi }_{TD3}(\text{s})=\text{arg}{max}_{\alpha }{Q}_{TD3}(s,a)$$

Here, S is the state representation, which includes the coverage hole information and other relevant variables) $\alpha$ is the action selected by the policy, and the ${Q}_{TD3}(s,a)$ does the TD3 algorithm and estimates the Q-value. The operation is iterative of detecting coverage holes and TD3 policy learning and coverage hole repair. At each time step t, the agent detects coverage holes, updates its policy, and takes action to repair the coverage hole. The process helps in improving the overall efficiency of the transmission of the data with less energy consumption. Once the coverage holes are repaired, then we perform the data transmission by UAV nodes. This optimal UAV node can be selected by using the CMO (Cat and Mouse Optimizer) Algorithm.

### Optimal UAV-relay selection

Each CH sends the data to the base station directly or selects the next CH for data transmission. In existing work, the next CHs are selected as a relay, which leads to instability in WSN. In addition, it takes much time to transmit the data due to choosing the next CH, but UAV-Relay directly transmits the data from CH to BS without any delay, which reduces energy consumption. Here, the edge-assisted it makes a huge difference in the optimization process, and the calculation of the buffer factor differs completely and. Edge-assisted UAVs typically have the leverage of edge computing resources to offload processing and to enhance their capabilities, which can show an impact on data transmission, coverage, energy consumption, and decision-making. The edge-assisted UAVs in this particular scenario have some important processes like Edge computing influence, Improved data transmissions for Buffer factor calculation and Dynamic Resource Allocation, and Adaptive Decisions. These are all the important aspects and advantages of edge-assisted UAVs. The optimal UAV selection after performing the CMO operation is ${u}^{*}$ Corresponding to the optimal solution, which can be expressed as

24 $${u}^{*} =\text{arg }{max}_{u}Fitness({P}_{m})$$

where ${P}_{m}$ represents the final position. The edge-assisted UAVs can access computing sources that are located at the edge of the network, which can improve decision-making and data processing capabilities. And also influences the calculation of the objective function. The fitness function evaluates how well a particular solution (in this case, the selection of a UAV) performs with respect to the objectives of the optimization problem. In the context of selecting the optimal UAV, the fitness function could be defined based on various criteria, such as minimizing energy consumption, maximizing coverage, and minimizing latency, To represent the mapping scenario between the CMO algorithm and UAV-relay selection Fitness Function, Decision Variables and Scheme are factors for selecting the UAV node,

The decision variables are the variables that the optimization algorithm can adjust to find the optimal solution. In the context of selecting the optimal UAV, decision variables could include parameters such as UAV location, UAV altitude, UAV transmission power, or other parameters that influence the UAV's performance and effectiveness in the network. The scheme refers to the overall approach or strategy used by the optimization algorithm to search for the optimal solution. In the case of CMO, which is inspired by the behavior of cats and mice, the scheme involves iteratively updating the positions of candidate solutions (UAVs) based on the behavior of cats (representing the optimal solution) and mice (representing potential solutions). The scheme includes mechanisms for exploration (searching for new solutions) and exploitation (refining promising solutions) to search the solution space efficiently.

The goal is to find the optimal configuration of UAV relays based on certain objectives or criteria (e.g., minimizing energy consumption, maximizing coverage).

The implementation of the CMO algorithm with the involvement of edge-assisted UAVs. The objective function with edge-assisted factors can defined as updated objective function *f(u),* which considers the edge-assisted factors like access to computing resources, data processing capabilities, and other relevant aspects of edge assistance.

The expression for the factors mentioned above can be expressed as

25 $$f\left(u\right)=\alpha .ResidualEnergy+ \beta . ExpectedCoverageRate\left(u\right)+\gamma .BufferFactor\left(u\right)+\delta .EdgeAssistedFactors\left(u\right)+\dots $$

where $\alpha ,\beta ,\gamma ,\delta ,etc$ are weighting factors. The edge-assisted factors that can show impact on the UAV performance, such as access to the edge resources and processing capabilities, and data transmission speed, these factors could be represented as functions that depend on the state of the UAV or the edge resources, for instance, let consider

26 $$\text{EdgeAssistedFactors}(\text{u}) =\zeta .\text{ EdgeResourceAvailability}(\text{u}) + \eta .\text{ ProcessingCapability}(\text{u})+ \dots $$

where the $\zeta \text{and }\eta$ are the weighing factors for edge resource availability and processing capability, respectively, the edge resource availability and process capability are based on the edge assistance it received. Some factors influence the performance, like latency resource allocation algorithms and other edge-assisted parameters. Hence, it selects the edge-assisted UAV as a relay that can move three-dimensional and better position to reduce energy consumption. This process reduces energy consumption and increases network lifetime. So, in the overall analysis, the CMO-based UAV selection involves a probabilistic approach to selecting the UAVs, and it considers parameters like link stability, connectivity, and energy as parameters for determining the Optimal UAV relay node.

## Simulation analysis

This section includes experimental findings, and it has two subsections, which include simulation setup and comparative analysis in comparison to many parameters such as Packet Delivery Ratio, Energy Consumption, Network Lifetime, Throughput, Delay, Reliability, Number of Alive Nodes, and Coverage Percentage.

### Simulation setup

The simulation setup of this model is briefly explained in this section. The simulation of this research is done by using an NS-3.26 network simulator executed in several steps like Quadtree clustering, Dynamic duty cycling, coverage hole detection and recovery, and UAV relay data transmission. The following Table 5 explains the simulation parameters.

**Table 5:** Simulation parameters of the proposed model.

Parameters	Values
Number of sensor nodes	100
Number of UAV nodes	5
Simulation area	500 × 500 ${\text{m}}^{2}$
Simulation distance	600 m
Modules	WIFI, Internet, IPV4
Initial energy of nodes	100 J
Packet interval	1.0 s
Number of rounds	100
Packet size	1024 bits
Transmission range	100 m
${\text{E}}_{\text{elec}}$	50 nj/bit
${\text{E}}_{amp}$	0.1pj/bit/m
${E}_{fs}$	Negligible
${E}_{mp}$	Varies

### Comparative analysis

In this section, a comparative analysis is performed between the proposed model and its contemporary protocols, and some initial conditions are taken from^13^ like initial energy of nodes and it is observed that this model has better performance when compared with the Coherent approach^5^, Repair algorithm^17^, and HWSN^18^. For all these protocols, the comparison is performed on different metrics such as Energy Consumption, Network Lifetime, Packet Delivery Ratio, Throughput, Delay, Reliability Number of Alive Nodes, and Percentage of Coverage.

#### Impact of energy consumption: ${\text{E}}_{\text{mp}}$

**Figure 5:**
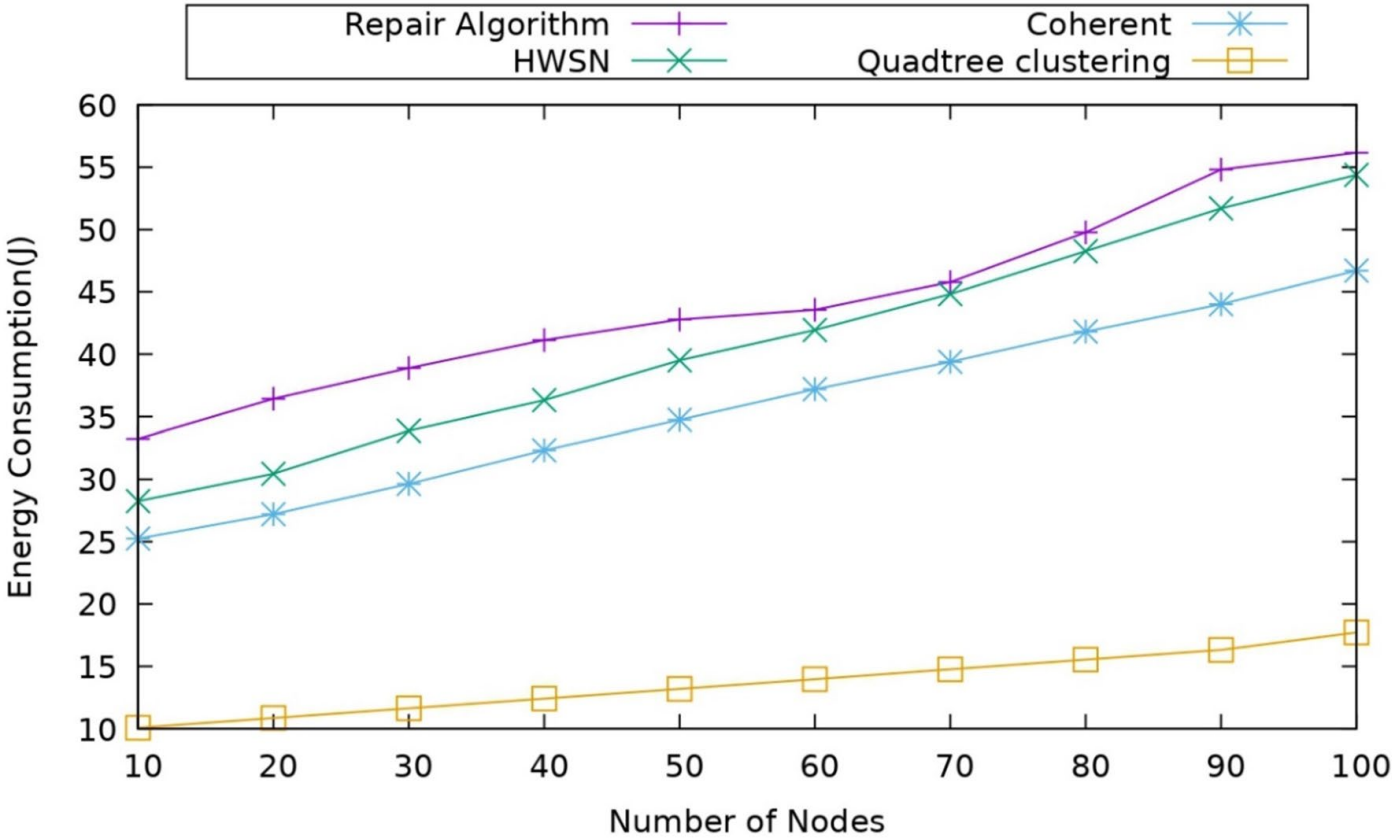
Comparison of energy consumption.

When it comes to the calculation of the energy, the energy difference between starting and current, which is known as residual energy, this metric is mainly used to analyze how the energy is consumed for performing all processes and estimating the overall energy consumed, and the energy consumption is represented as ${E}_{c}$

27 $${E}_{c}={N}_{i}-{E}_{r}$$

where ${N}_{i}$ represents the initial energy and ${E}_{r}$ represents the remaining energy. The comparison of energy consumption for different protocols is described in Fig. 5. While observing the outcomes, it is clear that the proposed model has better performance in this scenarioThe proposed method uses less energy in comparison to alternative approaches. This model uses a Quadtree structure, which efficiently manages the distribution of the sensor nodes according to the node density. So that the energy consumption during the clustering and routing is reduced, and the data is transmitted through UAV nodes, which helps in the energy consumption when compared to the coherent approach, it uses a mobile sink, which will reduce the efficiency in a way because it uses only one mobile sink. At the same time, the other sink node is static, which reduces the performance also. There is only a mobile sink, and the data traffic will increase for the sink node and, due to it, will depict its whole energy here. The coherent approach for clustering uses the MOEPO algorithm. In this model, we use the CMO algorithm for selecting the Optimal UAV relay node to transmit the data, which helps in making the energy consumption less. When it is observed, the proposed work energy consumption is coming under 20 J overall when the completion of the simulation time while we compare with the coherent approach where its energy consumption is almost 35–40 J while the completion of the simulation also the energy consumption of the repair algorithm is around 50 J for the whole simulation. By the above analysis, the proposed model achieves better performance.

#### Impact of network lifetime

**Figure 6:**
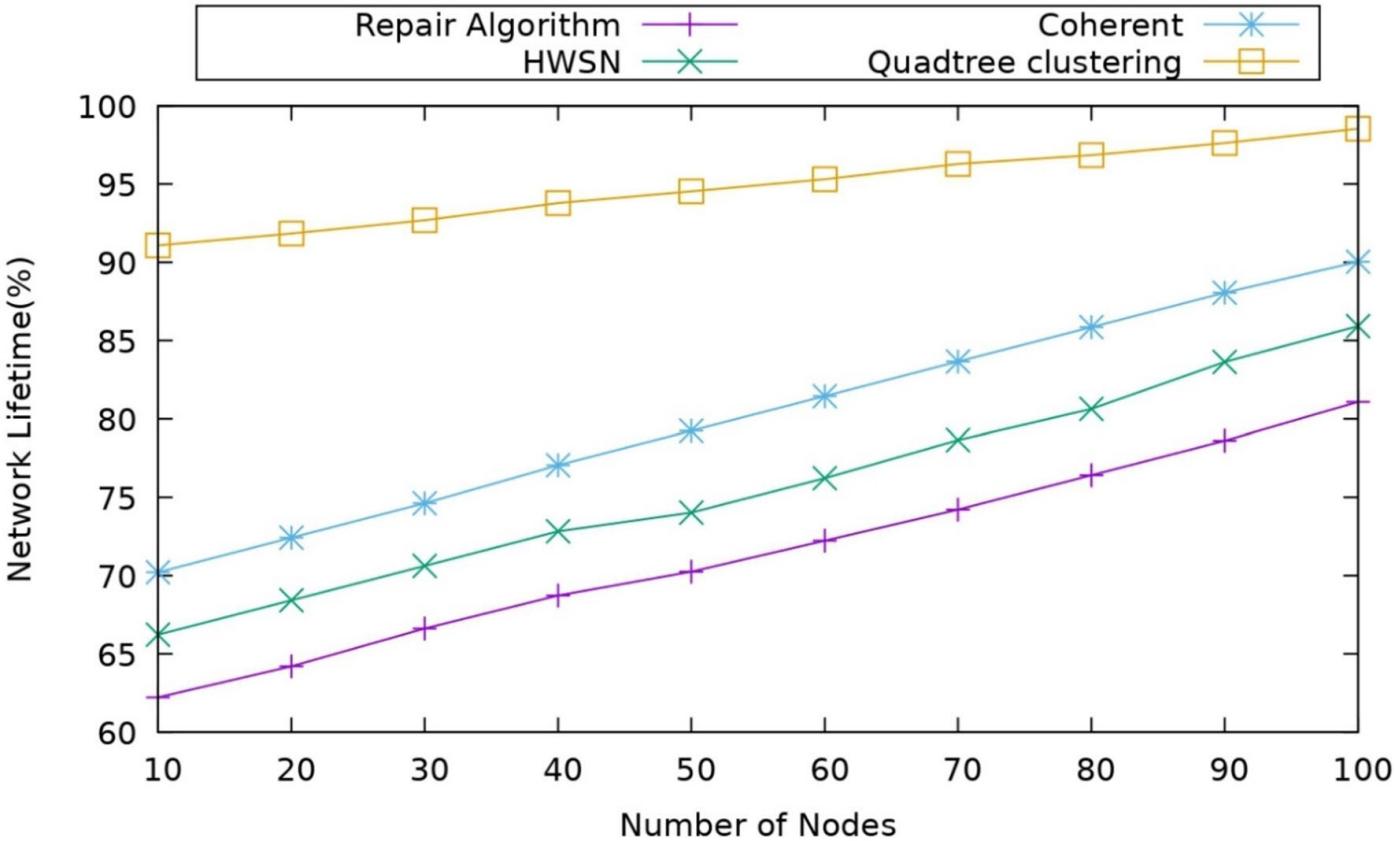
Comparison of network lifetime.

This metric main purpose is to assess the sensor network's overall lifetime, which is the calculation of the number of nodes that can survive after a particular time can be mentioned as a Network Lifetime. The comparison of the network lifetime to the number of nodes is shown in Fig. 6.

When compared with other protocols the proposed model is showing better performance in the terms of the network lifetime, The main added advantage in this model is that the data transmission is done by the UAV nodes so the transmission time will reduce also the packet loss so that the nodes energy can survive for more time also we consider duty cycling for the operation which will allow the nodes to make it sleep active and transmit the data which helps the node to manage the energy dissipation and the protocols repair algorithm concentrates on the coverage holes but it did not consider all the required parameters for the coverage hole detection which in this case it uses TA-TD3 algorithm and with two agents working for the coverage hole process where one agent is used for detecting the coverage hole while another agent is used for repairing the coverage hole which had added advantage to the model while comparing with its contemporary models like coherent approach or repair algorithm and also the lifetime is better than the existing models.

#### Impact of packet delivery ratio

**Figure 7:**
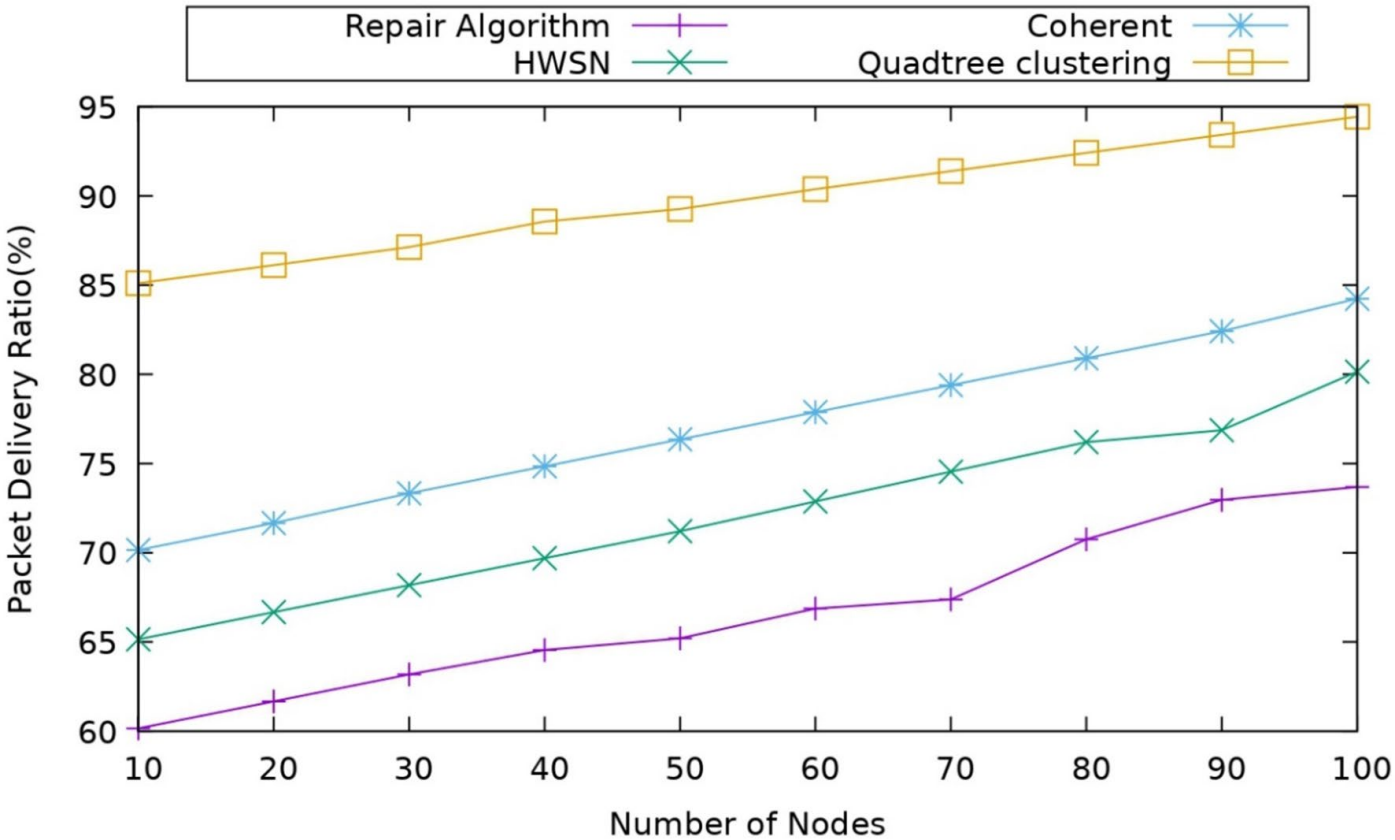
Comparison of packet delivery ratio.

Packet delivery ratio is the metric that is calculated as the ratio of the number of packets transmitted to the number of packets received. It can be represented as follows.

28 $$\text{PDR }= \frac{{P}_{R}}{{P}_{T}}$$

where ${P}_{R}$ represents the number of packets received and the ${P}_{T}$ are the number of packets transmitted. Figure 7 depicts the representation of the packet delivery for the proposed model and the existing models. As mentioned earlier, the proposed model uses UAV nodes as the relay nodes for data transmission, so the packet loss is reduced compared to the coherent model, which uses mobile sink nodes.

The optimal UAV nodes are selected based on the CMO algorithm, which helps in improving the data transmission. The optimal UAV node is selected based on factors like link stability, connectivity, and energy, which helps in choosing a UAV precisely for transmitting the data. As observed with its other protocols like coherent approach and Repair Algorithm, it is observed packet delivery ratio is very much improved here because this method avoids the hops. It directly transmits the data through the UAV nodes, which gives the added advantage when it is observed that the packet delivery ratio of the proposed model is nearly 85%. The coherent approach is around 70–75%, and the repair algorithm is around 65%, so by this, the proposed model outperforms the existing models.

#### Impact of throughput

**Figure 8:**
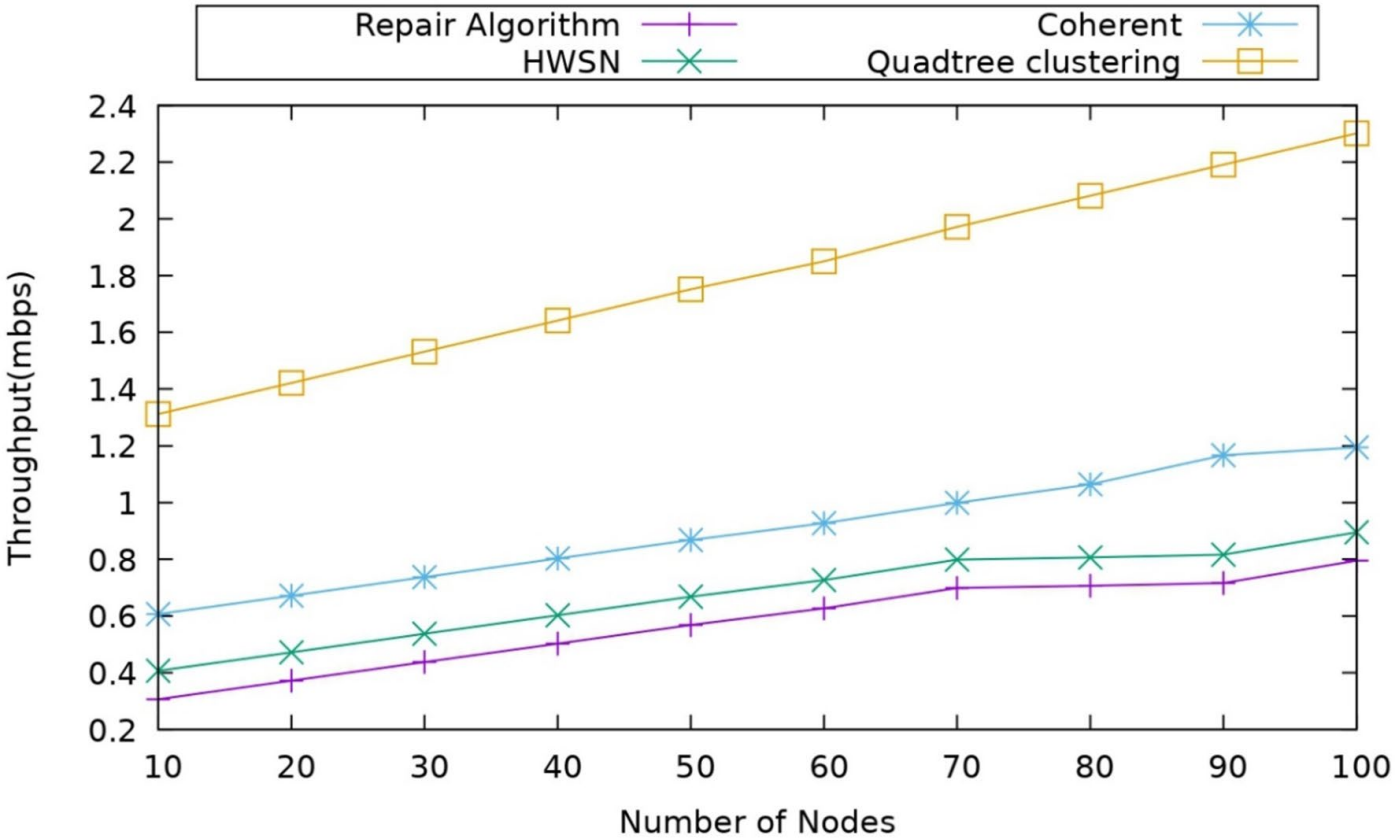
Comparison of throughput.

This metric mainly calculates the number of packets sent from source to destination over some time is measured as Throughput, and the throughput can be calculated as ${T}_{R}$ Which is defined as follows

29 $${T}_{R}=\frac{{S}_{t}}{t} \times 100$$

where the ${S}_{t}$ represents the number of packets transmitted successfully. Figure 8 represents the throughput for different existing models compared with the proposed model, and it is observed that the proposed model has more throughput.

The comparison results demonstrate that the proposed model outperforms the other existing models. The proposed model incorporated many like edge assisted UAVs, and it also performs dynamic duty cycling here. By using these assisted UAVs, unnecessary hops of transmission can be avoided, and hence, it reduces the packet loss with which the data transmission can become feasible. The existing works mainly depend on the static nodes as the relay nodes; however, the coherent approach had incorporated the mobile sink for data transmission, which improves the throughput, but still, it has only one mobile sink while the other sink node is static node so which will again show impact on the data which affects the Throughput. When it is observed that the repair algorithm and coherent approach achieved a throughput of around 0.5 to 1.2 MBPS, the proposed model had given a throughput of around 2.1 MBPS, which clearly indicates the model has better performance.

#### Impact of delay

**Figure 9:**
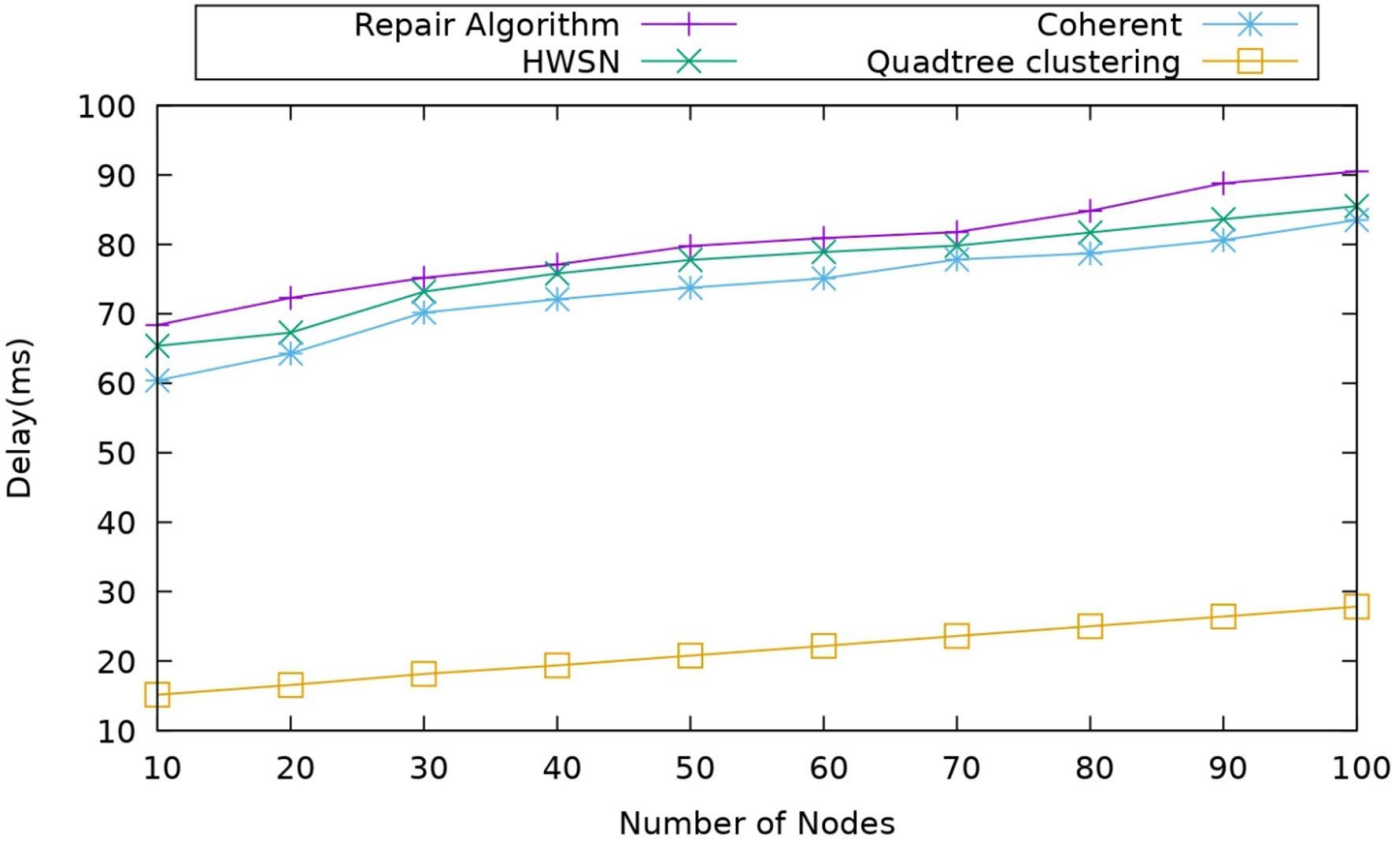
Comparison of delay.

This metric is used to calculate the additional time taken by the system to complete a process. When there is less delay, that indicates that the system has better performance and high efficiency of the system calculation of the delay can be calculated. ${D}_{L}$ Which is defined as follows

30 $${D}_{L}= {C}_{C}-{C}_{ex}$$

where ${C}_{C}$ is the current completion time, and the ${C}_{ex}$ is the expected completion time. When compared, the proposed model has less delay than the existing models. There are many reasons for the delay in the transmission, and Fig. 9 represents a comparison of the nodes and their delay for the proposed model as well as s and their delay for the proposed model as well as the existing models.

The main issue that can show an impact on the transmission and increase the delay is the coverage hole, which is basically an area of simulation where some nodes are left without any cluster. Due to this, the delay of transmission can take place, which is a major issue in the existing models; however, the proposed model uses the TA-TD3 algorithm, which helps in detecting the coverage holes and repairing them, so it will improve efficiency and reduce the delay. As we observed, the delay of the proposed model is around 10 to 25 ms. In comparison, the delay of the Coherent approach is around 60 ms, and this clearly indicates that the proposed model has less delay, which means more efficiency in the transmission.

#### Impact of reliability

**Figure 10:**
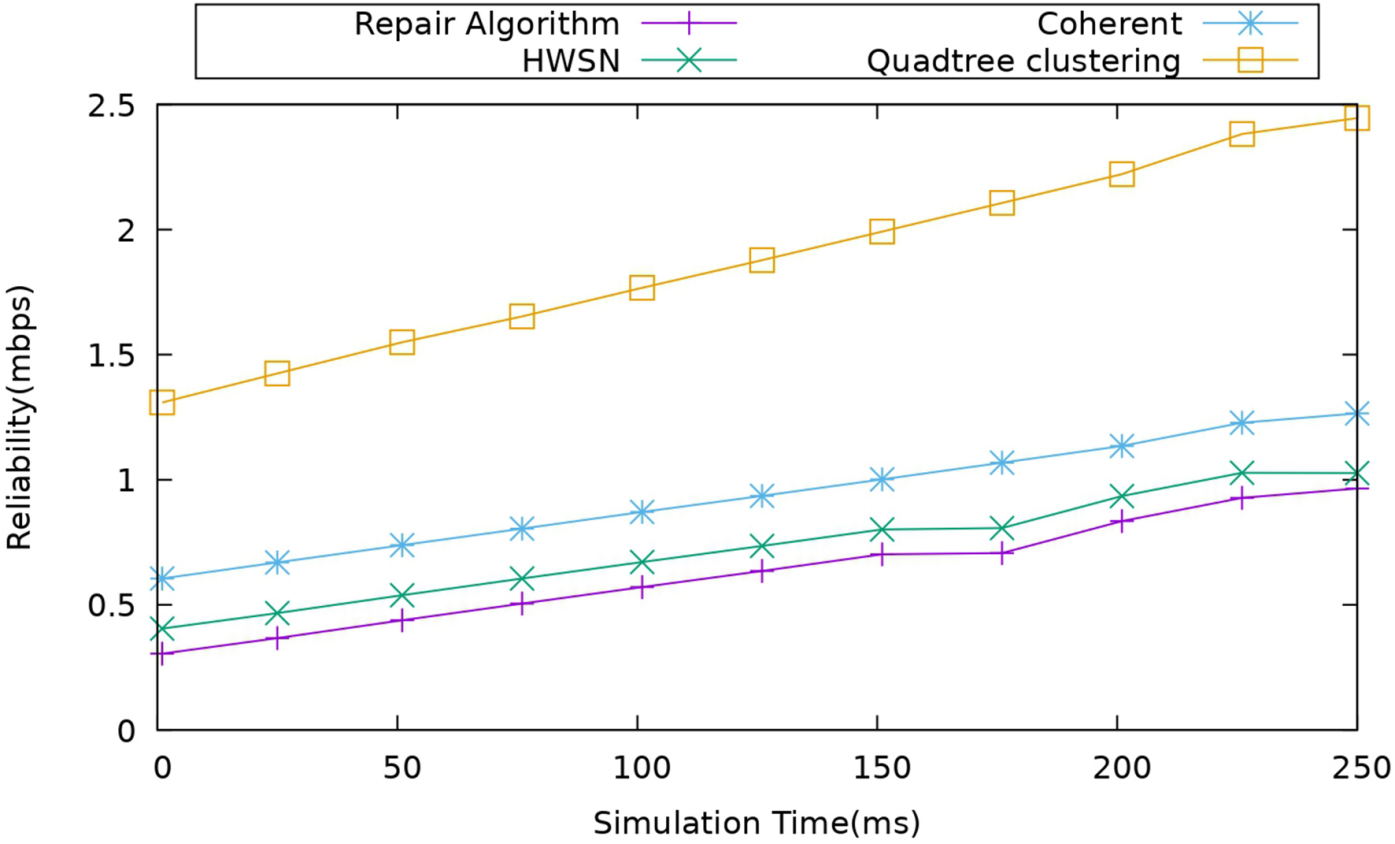
Comparison of reliability.

This metric in this scenario represents the ability of the deployed network to perform here. It includes the UAVs and ground nodes, and it is used to evaluate whether the model is consistently performing its intended functions when compared to the existing models. This model is more reliable in this scenario. It is measured in MBPS and below. Figure 10, it is represented.

Here, in this network model scenario, the reliability is represented in Mbps (Megabits per second) is a measure of the data transmission rate or throughput achieved by the simulated network. Here, the data in Mbps indicates how the data is transmitted successfully through a network. It’s a measure of the network’s capacity to deliver the data from source to destination in a given time frame. The data transmission is affected by many factors, and generally, the duty cycling and optimal edge-assisted UAVs provide more reliability and efficient data rates in this model.

#### Impact of number of alive node

**Figure 11:**
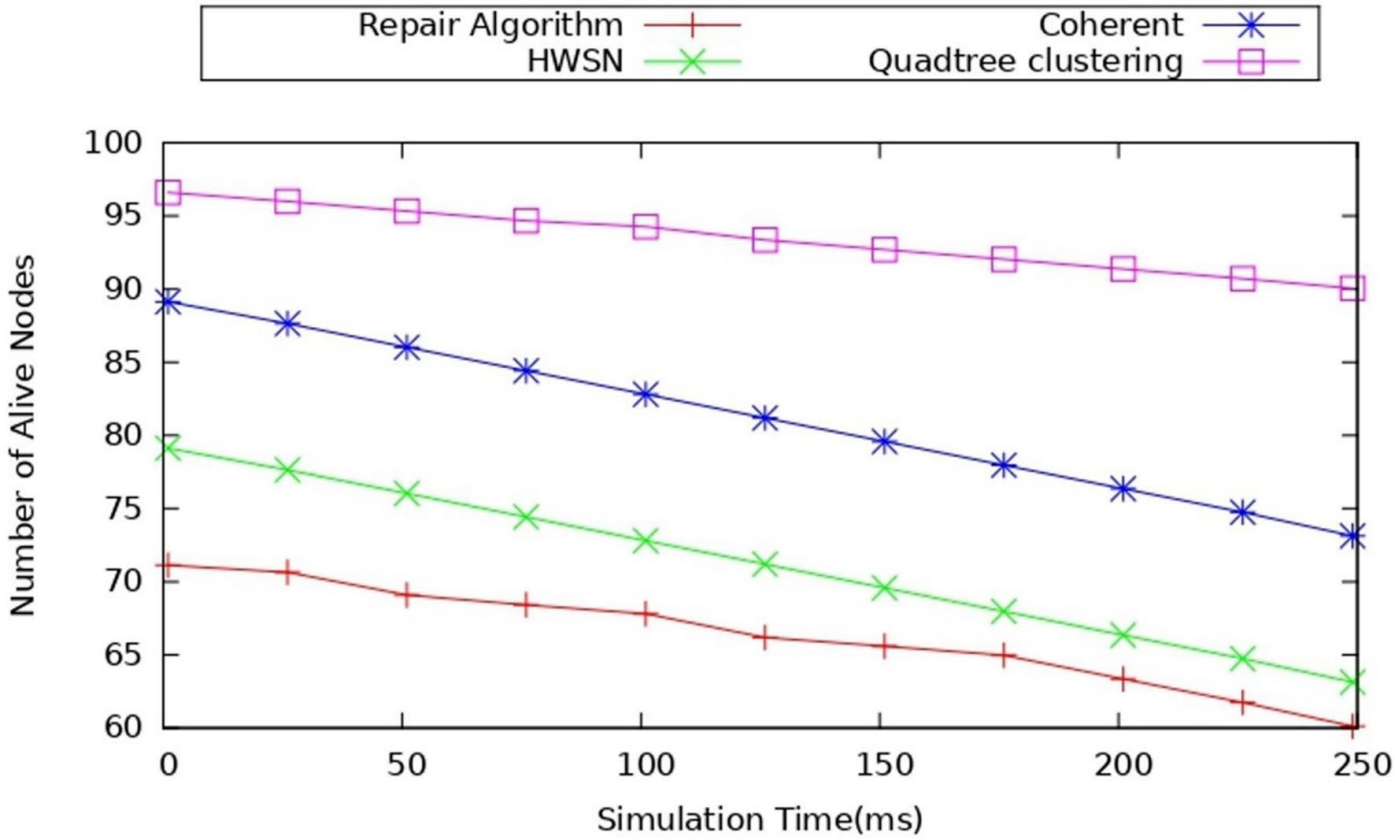
Comparison of the number of nodes alive.

This metric is used to calculate the count of the number of nodes alive after a certain time of simulation, which, in general, defines the node in an active state that is directly proportional to the network lifetime. Figure 11 represents the alive node comparison of the existing works. The alive nodes indicate how the network is performing, as we observed that the proposed model has more nodes alive after a certain simulation time. Also, we observed that as the simulation continued, the number of nodes alive was more when compared to existing models because it used the dynamic duty cycling model. Also, it uses the TA-TD3 algorithm, which helps in identifying the coverage hole and repairing it so the overall performance of the network will improve and reduces the energy consumption so the nodes will be more alive after many simulations time also when compared to the proposed model the alive nodes are minimized around 88 to 90 and the coherent approach the nodes have the nodes which are around 75 to 80 nodes and when compared with repair algorithm where the nodes are around 60–70 nodes alive around the simulation time when completed. When we observe the difference between the nodes is seen that there is a 10-node difference with the coherent approach and 20 20-node difference with the repair algorithm, and the above analyzation, it clearly indicates that the proposed model has more alive nodes after a certain simulation time.

#### Impact of percentage of coverage

**Figure 12:**
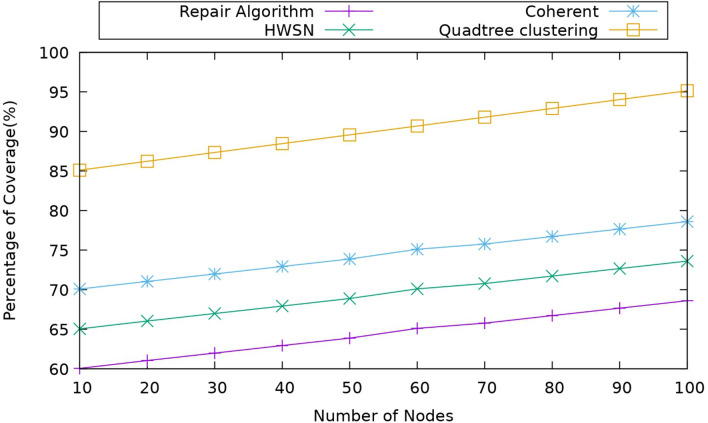
Comparison of percentage of coverage.

This metric is used to quantify the extent to which sensors cover a specific area or region. The percentage of coverage can be mathematically represented as the ratio of the area covered by sensors to the total area of interest in the percentage of coverage. ${P}_{c}$ can be expressed mathematically as

31 $${P}_{c}= \frac{{C}_{A}}{{T}_{A}}*100\%$$

where ${C}_{A}$ is represented as a coverage area and ${T}_{A}$ is represented as the total area; it means comparing the nodes of the proposed model with the existing models. Figure 12 below represents the comparison of the proposed model to the existing models, and it is observed that the area of coverage is greater for the proposed model. It is obtained because this model used the TA-TD3 algorithm to find out if any coverage holes are formed during the operation. This TA-TD3 algorithm made sure that no coverage holes are available, and if any coverage hole is detected, it repairs it with this, so due to this, the overall coverage is increased. Here, the proposed model coverage is around 85% to 90%. When the coherent approach coverage is around 70–75%, and the repair algorithm is around 60–65%, the usage of the coverage hole detection and recovery made a significant impact on the percentage of coverage of the nodes.

### Performance analysis

Our comparative analysis shows that while each of these studies contributes valuable insights into UAV-aided clustering and data collection in WSNs, our protocol offers a unique combination of advanced algorithms (IUKF for dynamic duty cycling, TA-TD3 for coverage hole detection and repair, and CMO for UAV relay selection) that collectively enhance energy efficiency, network longevity, and data transmission reliability.To validate the effectiveness of our protocol, we conducted extensive simulations comparing our approach with the mentioned UAV-aided clustering protocols. The results demonstrate significant improvements in energy consumption and network lifetime, supporting the robustness and efficiency of our proposed method.

**Figure 13:**
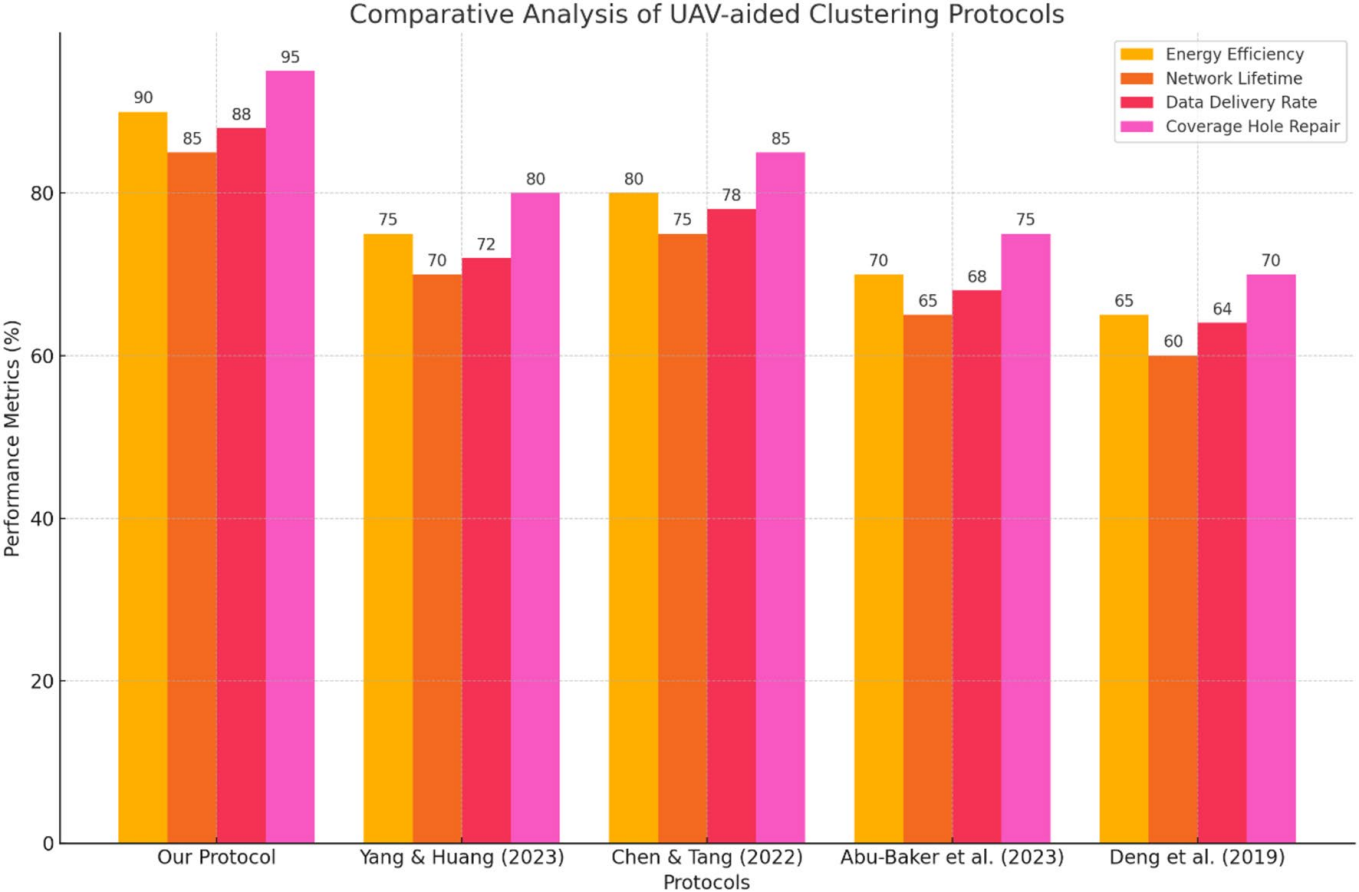
Comparison of UAV aided clustering protocols.

Here is a comparative analysis graph of UAV-aided clustering protocols is shown in Fig. 13, including our proposed protocol. The graph evaluates four performance metrics: energy efficiency, network lifetime, data delivery rate, and coverage hole repair efficiency. The protocols compared are: Our protocol^1,4,6^. When compared to parameters like Energy Efficiency, Our protocol demonstrates the highest energy efficiency at 90%, significantly outperforming the other protocols. and, Network Lifetime Our protocol also leads in improving network lifetime by 85%. Also when observed in Data Delivery Rate, Our protocol shows a high data delivery rate at 88%, surpassing the others. Also in Coverage Hole Repair, Our protocol achieves the highest coverage hole repair efficiency at 95%.

## Conclusion

- Initially, it creates a Network consisting of 100- Sensor nodes, 5- edge-assisted UAVs, and 1 base Station. Then, it performs the Quad-Tree Clustering process, in which the network is split into four Quad zones for clustering. Each zone is again divided into four Quadrants (four sections) based on the node density in the network, which improves the overall network performance. When it is observed in a coherent approach, it uses the K-Means clustering algorithm for clustering, but the challenge which is observed there is that the k values must be pre-defined, which affects the cluster and shows the impact on the stability of the network. Whereas when compared with the HWSN, where the nodes are deployed randomly based on calculating the redundant neighbors, it affects the operational phase time and reduces the efficiency. And in the coverage hole repair algorithm uses a maximum simple subnet to divide the network, which does not consider the existing conditions, which results in less efficient performance.
- After constructing clustering, it performs optimal CH selection by calculating the weight values of residual energy, node degree, node centrality, connectivity, and link stability. The high-weight value node is selected as the cluster head (CH). Others are known as cluster members (CM). Then, it performs cluster splitting and merging based on a threshold, which is calculated based on Cross-entropy by considering node density. When compared to the coherent approach, it considers the residual energy and distance for weight calculation. However, these parameters are not enough to select the cluster head efficiently because it might lead to poor cluster head selection, which increases the packet loss rate. In HWSN, there is no cluster head selection as it randomly selects the cluster head. In the repair algorithm, it chooses the cluster head based on the area that is designed by the polygon area. The parameters are not enough to consider, so while observing, the proposed model has the better performance in all the compared protocols.
- Next, it performs the Dynamic Duty Cycling process. In this, it reduces energy consumption. Here, dynamic duty cycling is based on the Improved Unscented Kalman Filter (IUKF) algorithm to schedule the sensor nodes into transmit, active, and sleep. This scheduling is performed by considering residual energy, expected coverage rate, buffer factor, and historical information of the nodes. This coherent approach uses a cross-entropy function for the cluster splitting and merging in order to maintain the performance, which has only limited advantage over the scenario. The repair algorithm depends on the non-connectivity of the network where existing networks and non-isolated nodes. Whereas in the HWSN, the nodes are self-healing nodes without outside coordination, which do not consider the global information, which leads to data collision, which leads to packet loss and reduces the efficiency.
- Next, it performs the Energy Efficient Coverage Hole detection and Recovery process. In this phase, it detects and recovers the coverage hole in the network. Here, it proposes a Twin Agent Based Twin Delay Deep Deterministic (TA-TD3) algorithm. The UAV detects the hole by TA-TD3. The coverage hole repair is performed by selecting the optimal node based on coverage level, mobility, and lifetime. As in the coherent approach, it uses the surveyor’s formula to detect the coverage holes, and it uses fuzzy logic for the coverage hole detection and recovery. However, the fuzzy parameters but the fuzzy parameters are not sufficient because they use only residual energy and stability. When it is observed in the repair algorithm, it uses two novel distributed algorithms that automatically detect/repair algorithms to restore connectivity. Still, the major drawback of this is that it only calculates holes based on only on the single-hop neighbors.
- Next, it performs the Optimal UAV-Relay Selection process. In this, it selects the Edge-assisted UAV as a relay that can move three-dimensional and in a better position to reduce energy consumption. The optimal UAV relay is selected based on the Cat and mouse-based optimization (CMO) algorithm by considering SNR, CSI, link stability, and channel gain. In an optimal UAV, the relay needs to adjust the flight position to maintain the stability of the transmission link. Whereas in the coherent approach, it calculates the next hop for transmitting the data if the direct relay is not available, and for selecting, it uses MO-EPO; however, it calculates by considering the radius, but it does not consider the transmitting power, which increases the latency for coverage hole detection which leads low network lifetime.

So, while comparing all the points mentioned above, the proposed model outperforms the existing model. Table 6 depicts the numerical analysis of the proposed and existing works, including average performance metrics values.

**Table 6:** Comparative Analysis of the proposed model with existing models.

Performance metrics	Repair algorithm	HWSN	Coherent approach	Quadree Clustering
Energy consumption (J)	45	55	42.5	18.4
Delay (ms)	87	89	83.5	35.5
Throughput (Mbps)	0.27	0.75	1.1	2.27
Packet delivery ratio (%)	68.5	75	82.5	87.5
Network lifetime (%)	82.5	86	90	95
Alive nodes	71	79	86.8	96
Reliability (Mbps)	0.73	0.65	1.15	2.25
Percentage of coverage (%)	63.5	67.5	76.5	95.5

## Futurescope

This research is mainly designed to use Edge-assisted UAVs in order to improve the performance of the network by building the network based on Quad-Tree clustering. As explained above, this model incorporates Four major processes building the Quad-tree Structure, which increases the network management capability and reduces the complexity. Then it performs the Dynamic duty cycling to schedule the nodes into operation, which reduces the data Collison, and then it performs the Coverage hole detection, which reduces the performance of the network, and then it repairs the coverage hole, and finally, it selects an optimal UAV relay node for data transmission from source to destination avoiding multi-hops transmission.

In future work, we plan to conduct a detailed analysis of the energy consumption of UAVs during their hovering phase. This will include:Empirical Measurements: Conducting experiments to measure the energy consumption of different UAV models while hovering in various environmental conditions.Simulation Models: Developing simulation models to predict the energy consumption based on UAV specifications and operational parameters.Optimization Strategies: Investigating strategies to minimize energy consumption during hovering, such as optimizing flight paths and using energy-efficient hardware components.This detailed analysis will provide valuable insights into the energy dynamics of UAVs and help in further optimizing their use in WSN applications".

The whole scenario of the proposed model is executed in the NS-3.26 simulator as a simulation environment, and many parameters are compared. The proposed model has shown better performance than existing models like the Coherent Approach, Repair Algorithm, and HWSN. In the Future, a security concept is planned to be introduced and incorporated using blockchain. This helps to prevent hackers from installing malicious nodes in the network and also improves data transmission because the data is encrypted and cannot be breached easily by hackers. This is going to be planned as the future scope of this work.

## Data Availability

All data generated or analyzed during this study are included in this published article.
